# Barriers and facilitators of effective health education targeting people with mental illness: a theory-based ethnographic study

**DOI:** 10.1186/s12888-018-1924-3

**Published:** 2018-10-30

**Authors:** N. F. Hempler, R. A. S. Pals, L. Pedersbæk, P. DeCosta

**Affiliations:** Diabetes Management Research, Steno Diabetes Center Copenhagen, Niels Steensens Vej 6, 2820 Gentofte, Denmark

**Keywords:** Health education, Qualitative research, Patient education, Psychosocial theories

## Abstract

**Background:**

Health education is particularly important for people with mental illness because they are at higher risk of becoming overweight or obese and developing type 2 diabetes than are members of the general population. However, little is known about how to provide health education activities that promote engagement and motivation among people with mental illness.

**Methods:**

This study used ethnographic methods to examine barriers and facilitators of effective health education targeting people with mental illness by applying the concept of flow as a theoretical framework. Flow refers to immersion in an activity and is related to motivation. Data were collected through participant observation during eight health-educating activities and were thematically analysed using the concept of flow. Fieldwork was carried out between May and July 2015 in Denmark.

**Results:**

Barriers to flow included: 1) information overload, particularly of biomedical rationales for behaviour change; 2) a one-size-fits-all approach that failed to address the needs and preferences of the target group; and 3) one-way communication allowing little time for reflection. Educators promoted a state of flow when they spoke less and acted outside of a traditional expert role, thus engaging participants in the activity. Flow was facilitated when educators were attentive and responsive to people with mental illness, and when they stimulated reflection about health and health behaviour through open-ended questions, communication tools and in small group exercises.

**Conclusions:**

This study suggests that more focus should be paid to training of educators in terms of skills to involve and engage people with mental illness in health education activities.

## Background

People with serious mental illness have a shorter life expectancy than do members of the general population [1–3]. Approximately 60% of this premature mortality is due to physical diseases such as cardiovascular disease and diabetes [4]. Several explanations for the excess mortality have been suggested, including unhealthy behaviours, adverse metabolic effects of antipsychotics, inadequate access to high-quality physical healthcare, and a cultural tendency to not consider physical diseases when treating mental illness [5–10].

Health behaviours are potentially modifiable. Previous research has shown that people with mental illness are as motivated as other members of the population to engage in health-promoting behaviours [11] However, eliciting behavioural change in people with mental illness has proven difficult; a review based on only 4 studies found some effect of lifestyle interventions on weight in people with psychotic disorders but no effect on blood pressure and cholesterol levels [12]. A recent study found no effect of a yearlong, intensive lifestyle coaching intervention targeting physical inactivity, unhealthy dietary habits and smoking, in people with schizophrenia [13]. This calls for rethinking and developing health education interventions focusing on educator behaviour to improve the treatment of people with mental illness. It has been suggested that lifestyle interventions within mental healthcare require tailoring to address the needs of people with mental illness and that further research is needed to explore barriers and facilitators for participation in health education activities from the perspective of people with mental illness [14–16].

People with mental illness report that the level of engagement and attitudes of educators play an important role in their motivation to engage in health behaviour change [14, 17–20]. For example, educators’ lack of motivation for physical activity can negatively affect the motivation of people with mental illness [20]. Another study supports incorporating the beliefs and perspectives on physical activity of people with mental illness into treatment for depression [17]. Educators report that patients’ poor state of health as well as a lack of cooperation with other groups of educators pose significant barriers to lifestyle changes [21]. Although existing literature addresses barriers and facilitators of health behaviour change among people with mental illness, little is known about how to facilitate health education activities that promote motivation for behaviour changes in this population. As traditional health education appears unsuccessful, it is relevant to explore alternative approaches, drawing on methods and models from behavioural science, using the experiences of the target group to inform and direct health education activities in practice. This study aims to identify barriers and facilitators of engaging health education across health education activities targeting people with mental illness, through multisided ethnographic fieldwork.

## Methods

### Theoretical framework

Flow refers to an inherently rewarding experiential state that can encourage a person to persist in and return to an activity, such as health behaviours; the theory of flow also focuses on interactionism, which highlights the importance of context [22, 23]. The concept of flow emerged from qualitative interviews about the nature of the experience when a particular activity goes well and is rooted in positive psychology [22, 24]. Flow research originates from examinations of intrinsic motivation, which is a key aspect in the self-determination theory. Intrinsic motivation occurs when an activity or behaviour is driven by an internal reward such as enjoyment, rather than being a means to obtain an external goal or a reward. [23]. According to Nakamura and Csikszentmihalyi, flow is a powerful motivating force [23]. When individuals are fully involved in an activity, they tend to find it enjoyable and intrinsically rewarding. Attention plays a key role in entering into and staying in a flow state.

The theory of flow focuses on the dynamic system composed of person and environment, thereby acknowledging the importance of context [23]. Motivation emerges in the interaction rather than being dictated by a pre-existing structure located within the individual or the environment [23]. Flow is associated with highly engaging activities and situations [22]. In a state of flow, thoughts, feelings and intentions are in harmony, and the activity itself is perceived as meaningful and associated with positive emotions. Activities that create flow tend to be remembered because they are associated with positive emotions and evaluated very positively by participants. Flow has also been associated with perceived competence: People with low perceived competence are likely to experience anxiety or boredom, depending on whether they value performing well at an activity. People with high perceived competence and efficacy are likely to report higher intrinsic motivation to perform an activity [25, 26].

Facilitators of flow include clear goals of activities, immediate feedback from educators and a challenge level that is just manageable for participants. An example of a barrier to flow is activity goals that focus on the needs of staff, e.g. reducing clinical risk and symptoms and improving medication adherence, rather than needs of individual participants [27]. In addition, long-term goals that are too easy or difficult for participants to manage may result in apathy, boredom, and anxiety [27]. Conditions in which flow is stimulated include:

- A balance between perceived skills and perceived challenges. When skills match challenges, participants’ attention is completely absorbed. If challenges exceed perceived skills, individuals typically become anxious; if perceived skills exceed challenges, they relax and may become bored.
- A clear set of goals that provides direction and purpose. The value of goals lies in channelling attention to structure the experience, rather than being ends in themselves.
- Clear and immediate feedback on progress. Immediate feedback allows individuals to make changes that improve activity-related performance [23, 24].

Flow was chosen as the theoretical framework in this study as it 1) addresses motivation and learning which is focus points in health education and 2) focuses on context; the interaction between educators and PMI as well as environment of importance for the delivery of health education.

### Data collection

Data collection was part of a larger study to develop a framework for a health education program targeting people with mental illness (PMI), based on a user-driven approach involving PMI, educators, and family members of PMI. The findings presented here represent the study’s initial phase, which focused on the identification of barriers and facilitators of effective health education among PMI. Insights from the fieldwork informed content and focus of workshops with PMI, educators, and family members which is reported elsewhere [28]. The larger study also involved professional development of 152 educators and testing of new methods in practice based on the developed framework (unpublished data).

We conducted multisided ethnographic fieldwork between May and July 2015 in Denmark. Ethnographic fieldwork studies groups and individuals in context, including interactions between people and their physical, material and institutional surroundings [29]. The fieldwork comprised participant observation of health-promoting activities in different care settings, including informal conversations with educators and PMI involved in the activities. Observations included 16 educators and 27 PMI in total. Health educating activities included individual lifestyle/health education consultations and group-based health education e.g. cooking classes, and physical activity.

Settings were identified in collaboration with health educators involved in the project. Educators at the identified municipal and regional care settings were contacted by e-mail or phone with a request to participate in the study. Inclusion of observation settings was based on geographical distribution, availability of individual and group-based activities, and municipal and regional outpatient care settings (Table 1). Inpatient clinics were excluded because the study excluded PMI who were hospitalised during the observation period. All activities targeted PMI in general, rather than individuals with specific diagnoses. One or two educators, such as social workers, nurses, and physiotherapists, facilitated each activity. Informed consent was obtained from participants before observations.

**Table 1:** Characteristics of fieldwork settings

Setting	Activity	Participants	Data collection
Outpatient clinic	Individual lifestyle consultation	1 professional 3 PMI	Observations of 3 consultations.
Community health center	Group-based cooking class	3 professionals 5 PMI	Observation. Informal interview
Community health center	Individual cooking class followed by a communal meal	3 professionals 4 PMI (1 PMI and 1 professional participated in the cooking class)	Observation. Informal interview
Community health center	Group-based exercise/physical activity	2 professionals (students) 4 PMI	Observation. Informal interview.
Community health center	Group-based education including a walk in the forest	3 professionals 5 PMI	Observation. Informal interview.
Day care center	Individual lifestyle consultation	1 professional 1 PMI	Observation.
Social home	No specific activity	2 professionals 4 PMI	Observation. Informal interview.
Outpatient clinic	Lifestyle consultation/screening	1 professional 1 PMI	Observation.

In Denmark, health activities targeting PMI, can be embedded in ambulatory psychiatry services and in the municipalities. However, format, approach and methods vary substantially from setting to setting. Also, general practitioners can play a role in terms of health behaviour and act as gatekeepers for PMI access to health activities.

### Observation

The aim of observations was to explore the activities, including the physical characteristics of social situations, and participation, dialogue and engagement among PMI, informed by the theoretical framework of flow. The themes were specified in an observation guide, and observations were documented in extensive field notes taken during activities. Themes comprised e.g. the role of the educator (facilitator, teacher or participant) and the role of PMI (proactive, active or passive) and were elaborated in the field notes. The observer participated in activities while maintaining the analytical and intellectual distance needed to interpret the social setting and record field notes [30]. The observers had a background in communication and public health science and were experienced with fieldwork in various health settings. No observers had work experience from psychiatry services. The participation of the observers was passive to moderate, depending on the activity [30].

### Data analysis

In order to explore the conditions in which health education activities were meaningful for the participants, the theory of flow was applied to the data analysis. We identified observable markers of flow including balance between challenges and skills, concentration, involvement and enjoyment. For example, a situation involved a dialogue between a PMI and an educator regarding daily life and food preferences; flow was noted as the PMI was engaged verbally (active participation by expressing and sharing needs and preferences with the educator) and non-verbally (concentrating and looking at the educator). Absence of flow included signs of apathy, boredom and anxiety. The analysis of barriers and facilitators of flow was concerned with forms of participation and the content of health-promoting activities. The analysis followed several steps: 1) extracting issues related to facilitators and barriers to flow by reading and re-reading observation notes; 2) analysis of the extracted data and identification of themes by writing down possible themes and categories, additional research questions, and ideas; and 3) interpretation of the data within each theme by identifying recurrent common and contrasting themes across observations. The categorization of themes was informed by the three conditions for flow as defined by Nakamura and Csikszentmihalyi; a balance between perceived skills and perceived challenges, a clear set of goals that provides direction and purpose, and clear and immediate feedback on progress [23].

Two of the authors, NFH and PD, independently coded the data and compared their interpretations, discussing any discrepancies until final agreement was reached. All authors participated in interpreting and discussing themes and categorizations of empirical material.

## Results

### Barriers to flow

#### Lack of balance between skills and challenges due to information overload

In many activities, communication was one-way; the educator did most of the talking and PMI were passive recipients of knowledge. Information overload often inhibited flow. This pattern occurred in both individual health education and group-based activities, such as cooking classes and walks. Educators dominated conversations with closed-ended questions about lifestyles and a strong focus on biomedical reasons for engaging in health behaviour. Educators offered solutions quickly and supported them with biomedical information about ‘cause and effect’. This is illustrated in the following excerpt from observation notes:

*At one point, the conversation turns to the fact that the PMI lacks vitamin D. The educator rationalises that this is linked to the fact that he ‘barricades’ himself indoors and thus does not get any daylight. The educator informs the PMI that there is something called a pineal gland, which produces melatonin. It needs sunlight. If he does not get it, he gets symptoms similar to depression. Therefore, he must at least go for a walk daily.* (Observation, individual lifestyle consultation)

PMI often had difficulty grasping educators’ specialised knowledge about triglycerides, hormones, carbohydrates and the like. Communicating knowledge was a central objective for educators, as articulated by one educator after a consultation:

*‘I think that I managed to communicate what I wanted to.’* (Observation, individual lifestyle consultation)

While some PMI displayed signs of boredom or apathy in response to information overload, some tried to interrupt the educator. One instance of this occurred during a nature-based education session that included a walk in the forest. The participants were not encouraged to share or reflect on their experiences with nature. At one point, in response to the educator showing a PowerPoint presentation on nature’s positive effect on mental health, a PMI interrupted to state:

*‘I cannot use all this theory. You are giving us too much information’. Another person adds: ‘We get the point. Getting out in nature is good for us. What you have just shown us is more appropriate for educators.’* (Observation, group education followed by a walk)

As exemplified here, the educator’s goal seemed to be communicating information, rather than allowing PMI to have and reflect on personal experiences of nature. Information overload was a reoccurring theme across activities and settings. Although the nature-based education session involved both classroom activities and a nature walk, the change of setting did not affect the educator’s approach. The educator dominated throughout the session.

#### Educator-defined goals

Many health education activities were organized under a comprehensive, structured plan. Although structure could provide direction for activities, a ‘one size fits all’ focus created a barrier to flow by channelling attention towards the plan rather than the needs of participants. In individual activities, programs, questionnaires, information and organisation of activities failed to take account of PMI individual characteristics or social backgrounds. Group-based activities demonstrated little focus on individual prerequisites, e.g. physical fitness.

Advice was often embedded in logical arguments, and educators told participants what they thought was good for them, a pattern that is antithetical to the experience of flow. A recurrent theme reflected the belief that increased knowledge about cause and effect of healthy lifestyle choices would elicit predetermined behavioural changes in PMI. One example was observed during a group education session in nature. Throughout the session, the educator focused squarely on communicating the positive effects of nature on health:

*‘Walking is good for the brain’ and ‘exercise will promote reflection.’* (Observation, group education followed by a walk)

Another example from observation of an individual lifestyle consultation:

*The educator is talking quite a lot, since there is an agenda to get through (the entire health form must be completed). The educator interrupts [the participant] once in a while to continue with the questions* (Observation, individual screening and lifestyle consultation)

The goal of most activities seemed predefined and static, with little room to adjust to participants’ current needs, feedback or personal interests.

#### One-way communication and little feedback

The focus of health education activities tended to be on the predefined agenda rather than on responses from PMI. Most educators did not encourage reflection or feedback from participants. This was evident in observations of both group-based and individual settings:

*When the educator integrates some stones into the teaching and passes them around, some participants start to share [personal stories] and show some interest. None of the educators ask questions or follow up on participants’ stories.* (Observation, group education followed by a walk)

Educators failed to acknowledge or address cues from participants and continued with the original agenda. Sometimes the educator addressed the concern with a rational solution, instead of exploring the issue further:

*The educator asks if PMI are familiar with the logo ‘nøglehulsmærket’ (a nutrition label stating that a food fulfils certain requirements for dietary fibre, saturated fat, sugar and salt). The PMI explains that he is familiar with it, but he does not know if he finds it trustworthy. The educator replies: ‘But it is [trustworthy].’ At the end of the session, the PMI again voices his concerns about ‘nøglehulsmærket’, to which the educator responds: ‘You can trust it’.* (Observation, individual health education and screening)

During one observation, a PMI explained that he experienced persistent stomach pain, nausea and vomiting. He raised this concern several times during the observation. The observer noted:

*It seems like his stomach problems weigh heavily on his mind and that this problem needs to be addressed before he will be able to work on any lifestyle changes.* (Observation, individual lifestyle consultation)

The cues from the PMI about his pain and nausea were not addressed. The educator continued the session as planned, assessed the PMI’s risk of diabetes, and gave advice on diet and lifestyle. Another example of participant engagement being overlooked by educators in favour of following a predefined plan is evident in the following observation:

*At one point the group stops at a bench. For the first time during the nature walk, everyone in the group is actively engaging in conversation and seems to be enjoying themselves. The educator interrupts: ‘We’ve got to get going, sorry’. A PMI responds: ‘I thought that the point [of the walk] was to talk to one another’.* (Observation, group education followed by a walk)

The need of educators to follow a pre-defined schedule rather than responding to cues and providing constructive feedback is illustrated in the following excerpt from observation notes:

*The educator asks if the patient would like to know his risk of diabetes. He says that he doesn’t believe that he has diabetes. The educator doesn’t respond to this but starts a screening test for diabetes. She [the educator] asks him a lot of questions. He scores 9 points, which indicates that he is at increased risk of type 2 diabetes. The educator then asks: ‘What are your thoughts about that? ‘He answers: ‘It doesn’t concern me’. She continues: ‘Would you like to know what you can do?’ The patient doesn’t reply. She continues by telling him what to be aware of, e.g. a potbelly.* (Observation, individual lifestyle consultation)

The educator’s attention was often not directed at the PMI. The primary purpose of feedback from the PMI could be interpreted as enabling the educator to complete required forms, rather than engaging the PMI in a dialogue about health practices:

*The educator is looking at her screen a lot and at the paper schedule lying on her desk. Several times she talks to the PMI while she is looking at the screen, so they do not always have eye contact* (Observation, individual lifestyle consultation).

### Facilitators of flow

#### Active involvement channelled attention in activities

Flow was observed when PMI were actively included in an activity and when the educator dominated less. During these times, less information was conveyed, and PMI were given an opportunity to focus on specific tasks. This created an opportunity for PMI-perceived skills to match with the challenge at hand, stimulating flow:

*The PMI is using her body actively during the cooking session. The PMI appears to concentrate and initiates many tasks on her own.* (Observation, individual cooking session)

Competition stimulated flow among some participants during a badminton session. However, the element of competition was not motivating for a woman who was unable to follow the counting of points:

*All participants are engaged [playing badminton], educators as well as PMI. A young male participant is especially committed during the competition where you can gain points. This has the opposite effect on a female participant who cannot keep up with the points.* (Observation, sports café)

As illustrated in this observation, maintaining a balance between challenges and skills was integral to sustaining flow in an activity.

#### Goals related to everyday life of participants

Flow was facilitated most frequently when PMI were actively involved in determining the direction of the activity. Health education activities that actively included PMI, such as cooking sessions and sports cafes, facilitated flow on occasion by engaging participants. Flow was also stimulated when educators engaged actively as equals in health-promoting activities. During individual sessions, flow was observed primarily in response to the educator asking open-ended questions concerning the PMI’s background and everyday life:

*At the end of the session, diet, smoking, alcohol and exercise are discussed in relation to the PMI’s work. This leads the PMI to open up and share details about his everyday life and what he likes. The PMI becomes more engaged.* (Observation, individual screening and lifestyle consultation)

#### Collective feedback from educators and participants

In the observed health-promoting activities, flow seemed to be stimulated when educators and participants were attentive and responded to other participants’ goals and related concerns. In a cooking session, educators asked open-ended questions about diet and weight loss, which encouraged dialogue among participants and educators:

*One of the educators contributes to this [dialogue] by asking open-ended questions of the participants. A participant talks about losing 13 kg. Another educator asks how, and the participant says that she has counted calories and been physically active. Another participant tells that she lost 15 kg without doing much. The educators praise the participants for having lost weight. A third participant expresses that she cannot accept that the medicine [she is taking] means that she does not feel full. This leads to a dialogue among the participants about medication, including how different medicines affect them.* (Observation, group-based cooking session)

Another example of collective feedback from participants occurred at the badminton session. The participants encouraged each other during the game, creating a positive and supportive environment:

*The dialogue is praising, acknowledging, and positive. This is also the case among participants. They encourage each other; especially [name of PMI] encourages [name of PMI] who has difficulties hitting the ball.* (Observation, sports café)

During the badminton session, the educators paid close attention to the participants, which also contributed to a supportive and positive environment. This is illustrated in the following excerpt from observation notes:

*A participant hurts his arm after 15 minutes. One of the educators leaves the session with him to do stretching exercises while the others continue to play.* (Observation, sports café)

## Discussion

Flow refers to an inherently rewarding experiential state that can encourage a person to persist in and return to an activity, such as health behaviours; the theory of flow also focuses on context. By using the concept of flow as a theoretical framework, this study sought to explore barriers and facilitators of effective health education targeting PMI, through multisided ethnographic fieldwork.

Information overload was a recurrent barrier to flow throughout the observations; the quantity and detailed nature of the information provided by educators indicated an imbalance between expected behavioural changes and the individual skills and resources of PMI. Educators often provided similar information based on a predefined goal that did not consider participants’ individual goals. The one-way communication approach and highly structured agenda did not allow time for reflection or understanding of PMI’s current motivation for health behaviour change. Educators continually overlooked cues from participants. Flow was observed more often when PMI were actively involved in the health-promoting activity. Active involvement was facilitated through open-ended questions related to PMIs’ skills and resources, dialogue, and constructive feedback. When PMIs were more engaged, educators provided less information overall, and the information provided was more likely to match the skills and resources of the PMI.

### Information overload is counterproductive

Behaviour change is more complex than merely providing advice on healthy lifestyles [31, 32]. The findings of this study indicate that advice on healthy lifestyles can be counterproductive if it is generalised or fails to take individuals’ motivations and resources into account. There is a risk that health education activities may become demotivating to PMI. In accordance with this finding, studies have shown that healthcare professionals may lack confidence in the ability of PMI to complete tasks and reach goals, which they find very discouraging [21, 33, 34].

The self-determination theory provides a useful framework for understanding the relationship between the provision of healthy lifestyle advice and individuals’ motivation for engaging in health behaviour change. Self-determination refers to an individual’s capacity to make choices that are the primary determinants of action [35]. This implies an experience of choice and decision-making directed at meeting self-selected goals. In contrast, an individual can experience choices as pressure to perform, e.g. to act in accordance with healthy lifestyle advice. The individual may not experience a sense of choice, leading to non-autonomous motivation for action. Health-promoting activities facilitated without consideration of individuals’ resources, skills and goals can be associated with feelings of guilt and shame if the recipients feel that they cannot comply with the recommended practices [36]. To address these negative consequences of non-autonomous motivation, Ryan and Deci [35] suggest that healthcare professionals help individuals explore their sense of choice and reflect on their reasons for action.

### One size does not fit all

Educators relied on a ‘one size fits all’ approach that undermined a focus on participants’ skills and resources and belied the complexity of health education. The ‘one size fits all’ approach can also be referred to as a biomedical ‘top-down’ approach in which educators are viewed as experts [37]. This approach contrasts with the ‘bottom-up’ approach, in which participants’ individual experiences and needs are considered. According to Jormfeldt [38], these paradigms co-exist and contradict each other within mental health care; a tendency exists to focus on measuring absence of symptoms (remission criteria), which is regarded as appropriate for evidence-based practice. However, remission criteria largely overlook patients’ subjective experiences that appear to be crucial to stimulating experiences of flow. In this study, the focus of educators on providing biomedical information prevented them from being curious about the individual needs and cues of the target group. Being sensitive and responsive to cues from PMI about their needs could have guided educators in directing the health education activity towards the concerns of PMI. This would have represented a bottom-up approach, acknowledging and attending to positive dimensions of health such as self-esteem, quality of life, and social relations [18, 27, 39, 40]. Bruun Jensen points to the same issue of contradictory paradigms in health education interventions. He posits a need to focus on a broad concept of health, embracing dimensions of a good life and social relations, rather than defining health as merely the opposite of disease [40].

### How can flow be promoted?

The experience of flow increases motivation to engage in a given activity [25, 26]. In this study, flow was observed in situations in which educators spoke less and operated outside the role of an expert. By tailoring advice or a specific health education activity to individuals’ interests and motivations, educators can encourage a state of flow to promote positive health behaviour among PMI. To do so, educators must move towards a more person-centred approach to health education. As health behaviour change necessitates active engagement with individuals’ values, goals and knowledge, a need arises for professionals to be more attentive and responsive to these issues [31]. Consistent with our findings, studies have shown that the facilitator style of educators is critical to engaging PMI [15, 31, 33]. This style includes educators’ behaviour during discussions, including showing openness and honesty and the choice of communication techniques and materials [33]. For example, interactive tools such as quizzes, small group tasks and the use of flipcharts/whiteboards can encourage attendance and stimulate reflection.

However, for health education activities to be truly democratic, PMI must have the opportunity to influence both the content and process of health education activities. Simovska refers to this as genuine participation and posits that it encourages development of personal meaning and allows PMI ownership of their learning processes [41]. Feedback from educators and other participants in a group activity can also facilitate flow and create a supportive environment. Group activities are an important social forum, allowing PMI to interact, feel a sense of belonging and gain support from other individuals in a similar situation [33]. However, our study showed that joining group activities can be a barrier to participating in health education activities for some PMI. The process of joining a new group can be eased if participants know each other and are organized into small groups. Another aspect concerns cognitive impairment and negative symptoms such as hallucinations, which may limit the benefit of the health intervention in PMI.

### Implications for health education practice

Many professionals agree with the philosophy of person-centred approaches to health education, but implementing these approaches in practice remains a challenge. On a structural level, a need exists to consider how health is defined and operationalised in the given context; e.g. are positive dimensions of health taken into account? Does the context allow for a broad definition of health, which is important in hardly-reached group such as PMI? Training and supervision of healthcare professionals’ communication skills could enhance the likelihood of stimulating intrinsic motivation of PMI in practice. Educator approaches and attitudes towards PMI are important to consider. Focus should be on the collaboration about health in a broad sense that incorporates PMI’s needs and views on their health and what is important to them. Techniques from motivational interviewing and recovery approaches such as personal goals may enable educators to better understand and support individual’s personal reasons for behaviour change and facilitate discussions [27, 42, 43]. The setting is also likely to play a role in supporting PMI. Lavie-Ajayi et al. point to general practitioners as key persons in relation to health behaviour change, due to a long-term and trusting relationship with PMI [44]. Although this study focused on PMI, the findings may be transferable to other hardly-reached groups and PMI populations in other domains of rehabilitation and community integration not focusing on health.

### Strengths and limitations

A key strength of this study is its qualitative approach. The application of the theoretical framework of flow and ethnographic methods provided an in-depth understanding of the issues that facilitate and impede flow for PMI in health education activities. The framework sheds light on the interaction. Additionally, the large number and geographical dispersion of study sites are strengths. A limitation of the study is that we were able to conduct observations in only a limited number of sessions at each site. It would be beneficial to follow health education activities over a longer time period. Including the perspectives of PMI regarding facilitators and barriers to the experience of flow would also strengthen the findings. However, it was not possible to conduct formal interviews.

It is also possible that PMI are attentive but not in a state of flow, which observations might not be able to uncover. Also, it is not certain that observers always were able to detect flow when it occurred. To reach saturation for the identified themes, we documented observations in extensive field notes and analysed data using a validated approach relying on continuous monitoring and conceptualisation of data [45].

## Conclusion

This study sought to identify barriers and facilitators of flow in health education settings. A focus on flow could act as a starting point to improve health education targeting PMI. Instead of focusing on a specific activity more attention should be paid to the way activities are shaped and conducted that allow a sense of flow e.g. how to create a balance between goals and challenges, how to deliver feedback and how to create a playful environment. Creating flow is in line with an individualised approach, person-centeredness and a bottom-up approach. Promoters of flow in our study included attentiveness and responsiveness to individuals’ values, goals, knowledge and everyday life. Several barriers were also identified suggesting that more focus should be paid to training of educators to promote flow in the educational setting.

## Abbreviation

PMI: People with mental illness
